# 4-Methyl­benzyl 4-amino­benzoate

**DOI:** 10.1107/S1600536810007075

**Published:** 2010-03-03

**Authors:** Ali Haider, Zareen Akhter, Mohammad Saif Ullah Khan, Michael Bolte, Humaira M. Siddiqi

**Affiliations:** a Department of Chemistry, Quaid-I-Azam University, Islamabad 45320, Pakistan; b Institut für Anorganische Chemie, J. W. Goethe-Universität Frankfurt, Max-von-Laue-Strasse 7, 60438 Frankfurt/Main, Germany

## Abstract

The dihedral angle between the two benzene rings in the title compound, C_15_H_15_NO_2_, is 65.28 (12)°. The crystal structure is stabilized by N—H⋯N and N—H⋯O hydrogen bonds, leading to the formation of supra­molecular chains along the *a*-axis direction.

## Related literature

For the reduction of aryl-nitro compounds, see: Tafesh & Weiguny (1996 ▶); Vass *et al.* (2001 ▶); Entwistle *et al.* (1977 ▶); Bavin (1958 ▶); Yuste *et al.* (1982 ▶); Idrees *et al.* (2009 ▶). For the uses of amines, see: Kumarraja & Pitchumani (2004 ▶).
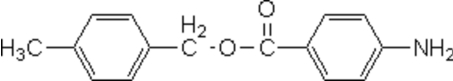


## Experimental

### 

#### Crystal data

C_15_H_15_NO_2_
*M* *_r_* = 241.28
Monoclinic, 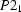

*a* = 8.2097 (12) Å
*b* = 5.5344 (5) Å
*c* = 14.293 (2) Å
β = 98.531 (12)°
*V* = 642.24 (14) Å^3^
*Z* = 2
Mo *K*α radiation
μ = 0.08 mm^−1^
*T* = 173 K
0.27 × 0.13 × 0.13 mm

#### Data collection

Stoe IPDSII two-circle diffractometer
4021 measured reflections
1322 independent reflections
960 reflections with *I* > 2σ(*I*)
*R*_int_ = 0.093

#### Refinement

*R*[*F* ^2^ > 2σ(*F* ^2^)] = 0.043
*wR*(*F* ^2^) = 0.094
*S* = 0.91
1322 reflections
173 parameters
1 restraint
H atoms treated by a mixture of independent and constrained refinement
Δρ_max_ = 0.14 e Å^−3^
Δρ_min_ = −0.13 e Å^−3^

### 

Data collection: *X-AREA* (Stoe & Cie, 2001 ▶); cell refinement: *X-AREA*; data reduction: *X-AREA*; program(s) used to solve structure: *SHELXS97* (Sheldrick, 2008 ▶); program(s) used to refine structure: *SHELXL97* (Sheldrick, 2008 ▶); molecular graphics: *XP* (Sheldrick, 2008 ▶); software used to prepare material for publication: *SHELXL97*.

## Supplementary Material

Crystal structure: contains datablocks I, global. DOI: 10.1107/S1600536810007075/tk2629sup1.cif

Structure factors: contains datablocks I. DOI: 10.1107/S1600536810007075/tk2629Isup2.hkl

Additional supplementary materials:  crystallographic information; 3D view; checkCIF report

## Figures and Tables

**Table 1:** Hydrogen-bond geometry (Å, °)

*D*—H⋯*A*	*D*—H	H⋯*A*	*D*⋯*A*	*D*—H⋯*A*
N1—H1*A*⋯O2^i^	0.88 (4)	2.12 (5)	2.977 (4)	164 (4)
N1—H1*B*⋯N1^ii^	0.96 (6)	2.37 (6)	3.278 (3)	158 (4)

Symmetry codes: (i) 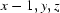; (ii) 
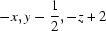
.

**Fig. 1.**
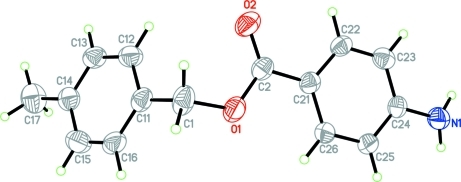
Perspective view of (I) with the atom numbering scheme. The displacement ellipsoids are at the 50% probability level and H atoms are drawn as small spheres of arbitrary radii.

## supplementary crystallographic information

### Comment

Reduction of aryl-nitro compounds to their corresponding amines is an important chemical transformation in synthetic organic chemistry mainly due to the fact that the amino group can serve as the site for further derivatization (Tafesh *et al.*, 1996; Vass *et al.*, 2001). Amines are important intermediates in the production of many pharmaceuticals, photographic materials, agrochemicals, polymers, dyes, and rubber materials (Kumarraja & Pitchumani, 2004). Selective reduction nitro-aromatics to amines can be achieved by hydrogen transfer using Pt—C (Entwistle *et al.*, 1977), Pd—C (Bavin *et al.*, 1958) and Raney Ni (Yuste *et al.*, 1982) catalysts. Most commonly applied or reported methods are direct catalytic hydrogenation and catalytic hydrazine reduction. The reduction of 1,4-bis(4-nitrobenzoyloxymethyl) benzene has been carried out using the catalytic hydrogenation method. It is important to note that the process requires much care in the addition of hydrazine, in order to prevent the breakdown of the ester linkage, as hydrazides may be formed from carboxylic esters in the absence of the catalyst or even if the catalyst is not properly charged (Idrees *et al.*, 2009). The limited addition of the hydrazine in the presence of activated catalyst can also cause the breakage of ester linkage not from the aryl carbon but from the acyl carbon as proved by the crystal structure of the title compound, (I). Herein, the synthesis and the crystal structure of (I) are reported.

The dihedral angle between the two benzene rings in (I) is 65.28 (12)°. The crystal structure is stabilized by N—H···N and N—H···O hydrogen bonds, Table 1, which lead to supramolecular chains along the *a* direction.

### Experimental

Compound (I) was synthesized in two steps. In the first step, a mixture of 1,4-bis(chloromethyl)benzene Aldrich; 2.00 g, 0.0114 mol), anhydrous K_2_CO_3_ (3.154 g, 0.0229 mol) and 4-nitrobenzoic acid (3.824 g, 0.0229 mol) were added to a two neck round bottom flask charged with DMF (50 ml). This was heated at 393 K for 12 h under an nitrogen atmosphere. After cooling to room temperature, the reaction mixture was poured into water (800 ml) to precipitate a yellow solid which was washed thoroughly with water and then separated by filtration. In the second step a 250 ml two neck flask was charged with the just synthesised yellow solid (1.00 g, 2.84 mmol) and was refluxed in ethanol with 5% palladium on carbon (Pd/C, 0.06 g), followed by the drop-wise addition of hydrated hydrazine (80%) diluted in ethanol. The mixture was refluxed for 8 h and then filtered to remove Pd/C. The solvent was evaporated and the resulting crude solid was recrystallized from ethanol to afford crystals (yield:68%, m.pt.: 397 K).

### Refinement

Hydrogen atoms bonded to C were included in calculated positions [C—H = 0.95–0.99 Å] and refined as riding [*U_iso_*(H) = 1.2–1.5*U_eq_*(C)]. The H atoms bonded to N were isotropically refined. Due to the absence of anomalous scatterers, the absolute structure could not be determined and 773 Friedel pairs were merged.

### Crystal data

**Table:**

C_15_H_15_NO_2_	*F*(000) = 256
*M**_r_* = 241.28	*D*_x_ = 1.248 Mg m^−^^3^
Monoclinic, *P*2_1_	Mo *K*α radiation, λ = 0.71073 Å
Hall symbol: P 2yb	Cell parameters from 2492 reflections
*a* = 8.2097 (12) Å	θ = 4.0–25.9°
*b* = 5.5344 (5) Å	µ = 0.08 mm^−^^1^
*c* = 14.293 (2) Å	*T* = 173 K
β = 98.531 (12)°	Prism, colourless
*V* = 642.24 (14) Å^3^	0.27 × 0.13 × 0.13 mm
*Z* = 2	

### Data collection

**Table:**

Stoe IPDSII two-circle diffractometer	960 reflections with *I* > 2σ(*I*)
Radiation source: fine-focus sealed tube	*R*_int_ = 0.093
graphite	θ_max_ = 25.7°, θ_min_ = 3.5°
ω scans	*h* = −9→9
4021 measured reflections	*k* = −6→5
1322 independent reflections	*l* = −17→17

### Refinement

**Table:**

Refinement on *F*^2^	Secondary atom site location: difference Fourier map
Least-squares matrix: full	Hydrogen site location: inferred from neighbouring sites
*R*[*F*^2^ > 2σ(*F*^2^)] = 0.043	H atoms treated by a mixture of independent and constrained refinement
*wR*(*F*^2^) = 0.094	*w* = 1/[σ^2^(*F*_o_^2^) + (0.0405*P*)^2^] where *P* = (*F*_o_^2^ + 2*F*_c_^2^)/3
*S* = 0.91	(Δ/σ)_max_ = 0.028
1322 reflections	Δρ_max_ = 0.14 e Å^−^^3^
173 parameters	Δρ_min_ = −0.13 e Å^−^^3^
1 restraint	Extinction correction: *SHELXL97* (Sheldrick, 2008), Fc^*^=kFc[1+0.001xFc^2^λ^3^/sin(2θ)]^-1/4^
Primary atom site location: structure-invariant direct methods	Extinction coefficient: 0.077 (11)

### Special details

**Table:**

Geometry. All esds (except the esd in the dihedral angle between two l.s. planes) are estimated using the full covariance matrix. The cell esds are taken into account individually in the estimation of esds in distances, angles and torsion angles; correlations between esds in cell parameters are only used when they are defined by crystal symmetry. An approximate (isotropic) treatment of cell esds is used for estimating esds involving l.s. planes.
Refinement. Refinement of *F*^2^ against ALL reflections. The weighted *R*-factor wR and goodness of fit *S* are based on *F*^2^, conventional *R*-factors *R* are based on *F*, with *F* set to zero for negative *F*^2^. The threshold expression of *F*^2^ > σ(*F*^2^) is used only for calculating *R*-factors(gt) etc. and is not relevant to the choice of reflections for refinement. *R*-factors based on *F*^2^ are statistically about twice as large as those based on *F*, and *R*- factors based on ALL data will be even larger.

### Fractional atomic coordinates and isotropic or equivalent isotropic displacement parameters (Å^2^)

**Table:**

	*x*	*y*	*z*	*U*_iso_*/*U*_eq_	
N1	0.0100 (4)	0.0360 (6)	0.9396 (2)	0.0442 (8)	
H1A	−0.086 (5)	0.090 (9)	0.911 (3)	0.057 (12)*	
H1B	0.007 (7)	−0.130 (11)	0.958 (3)	0.090 (17)*	
O1	0.5634 (3)	0.6085 (5)	0.77036 (17)	0.0454 (6)	
O2	0.7240 (3)	0.3098 (5)	0.83952 (16)	0.0468 (7)	
C1	0.7064 (5)	0.7251 (7)	0.7398 (3)	0.0475 (9)	
H1C	0.8064	0.6833	0.7845	0.057*	
H1D	0.6922	0.9027	0.7412	0.057*	
C2	0.5874 (4)	0.4043 (6)	0.8206 (2)	0.0347 (8)	
C11	0.7290 (4)	0.6485 (6)	0.6420 (2)	0.0383 (8)	
C12	0.8155 (4)	0.4400 (6)	0.6259 (2)	0.0414 (9)	
H12	0.8593	0.3410	0.6779	0.050*	
C13	0.8388 (4)	0.3743 (6)	0.5354 (2)	0.0405 (9)	
H13	0.8994	0.2321	0.5265	0.049*	
C14	0.7755 (4)	0.5117 (6)	0.4576 (2)	0.0405 (8)	
C15	0.6888 (5)	0.7204 (7)	0.4742 (3)	0.0511 (11)	
H15	0.6446	0.8195	0.4224	0.061*	
C16	0.6659 (4)	0.7853 (7)	0.5643 (3)	0.0464 (9)	
H16	0.6053	0.9276	0.5733	0.056*	
C17	0.7999 (6)	0.4368 (9)	0.3599 (3)	0.0601 (12)	
H17A	0.6988	0.3612	0.3278	0.090*	
H17B	0.8909	0.3210	0.3639	0.090*	
H17C	0.8258	0.5792	0.3241	0.090*	
C21	0.4371 (4)	0.3093 (6)	0.8495 (2)	0.0337 (8)	
C22	0.4413 (4)	0.0941 (6)	0.9016 (2)	0.0345 (8)	
H22	0.5425	0.0099	0.9172	0.041*	
C23	0.3008 (4)	0.0031 (6)	0.9306 (2)	0.0376 (8)	
H23	0.3064	−0.1430	0.9659	0.045*	
C24	0.1503 (4)	0.1226 (6)	0.90885 (19)	0.0321 (7)	
C25	0.1451 (4)	0.3382 (7)	0.8578 (2)	0.0373 (8)	
H25	0.0437	0.4225	0.8429	0.045*	
C26	0.2854 (4)	0.4302 (6)	0.82882 (19)	0.0352 (8)	
H26	0.2796	0.5774	0.7943	0.042*	

### Atomic displacement parameters (Å^2^)

**Table:**

	*U*^11^	*U*^22^	*U*^33^	*U*^12^	*U*^13^	*U*^23^
N1	0.0384 (18)	0.047 (2)	0.0480 (17)	−0.0004 (15)	0.0081 (14)	0.0099 (15)
O1	0.0416 (14)	0.0390 (13)	0.0604 (14)	0.0023 (12)	0.0232 (11)	0.0028 (12)
O2	0.0303 (13)	0.0573 (16)	0.0533 (14)	0.0020 (13)	0.0077 (11)	−0.0018 (13)
C1	0.048 (2)	0.036 (2)	0.064 (2)	−0.0084 (18)	0.0270 (18)	−0.0041 (17)
C2	0.0344 (19)	0.0332 (18)	0.0375 (16)	0.0003 (16)	0.0084 (14)	−0.0069 (15)
C11	0.0316 (18)	0.0346 (19)	0.0508 (18)	−0.0047 (15)	0.0133 (14)	−0.0020 (15)
C12	0.042 (2)	0.040 (2)	0.0413 (16)	0.0086 (17)	0.0056 (14)	0.0014 (15)
C13	0.040 (2)	0.0333 (19)	0.0489 (19)	0.0037 (15)	0.0086 (15)	−0.0030 (15)
C14	0.037 (2)	0.038 (2)	0.0455 (17)	−0.0061 (17)	0.0059 (15)	−0.0006 (16)
C15	0.044 (2)	0.047 (2)	0.060 (2)	−0.0007 (19)	0.0001 (18)	0.0148 (18)
C16	0.040 (2)	0.0305 (18)	0.073 (2)	0.0047 (17)	0.0207 (17)	0.0053 (18)
C17	0.067 (3)	0.069 (3)	0.0439 (19)	−0.011 (2)	0.0063 (18)	−0.004 (2)
C21	0.0356 (18)	0.0357 (18)	0.0300 (14)	0.0023 (16)	0.0059 (13)	−0.0047 (14)
C22	0.0321 (18)	0.0365 (18)	0.0349 (15)	0.0051 (16)	0.0054 (13)	−0.0051 (14)
C23	0.043 (2)	0.0371 (18)	0.0314 (16)	0.0012 (17)	0.0011 (14)	−0.0023 (13)
C24	0.0346 (18)	0.0328 (17)	0.0295 (14)	−0.0026 (16)	0.0069 (13)	−0.0048 (14)
C25	0.0319 (17)	0.045 (2)	0.0349 (16)	0.0060 (17)	0.0061 (13)	0.0029 (15)
C26	0.039 (2)	0.0351 (18)	0.0328 (15)	0.0049 (16)	0.0090 (14)	0.0021 (14)

### Geometric parameters (Å, °)

**Table:**

N1—C24	1.378 (5)	C14—C17	1.499 (5)
N1—H1A	0.88 (4)	C15—C16	1.377 (5)
N1—H1B	0.96 (6)	C15—H15	0.9500
O1—C2	1.338 (4)	C16—H16	0.9500
O1—C1	1.462 (4)	C17—H17A	0.9800
O2—C2	1.230 (4)	C17—H17B	0.9800
C1—C11	1.499 (5)	C17—H17C	0.9800
C1—H1C	0.9900	C21—C22	1.402 (5)
C1—H1D	0.9900	C21—C26	1.406 (4)
C2—C21	1.457 (5)	C22—C23	1.378 (5)
C11—C16	1.379 (5)	C22—H22	0.9500
C11—C12	1.392 (5)	C23—C24	1.395 (5)
C12—C13	1.384 (5)	C23—H23	0.9500
C12—H12	0.9500	C24—C25	1.396 (5)
C13—C14	1.383 (5)	C25—C26	1.378 (5)
C13—H13	0.9500	C25—H25	0.9500
C14—C15	1.395 (5)	C26—H26	0.9500
			
C24—N1—H1A	118 (3)	C15—C16—C11	121.5 (3)
C24—N1—H1B	119 (3)	C15—C16—H16	119.3
H1A—N1—H1B	113 (5)	C11—C16—H16	119.3
C2—O1—C1	118.2 (3)	C14—C17—H17A	109.5
O1—C1—C11	111.7 (3)	C14—C17—H17B	109.5
O1—C1—H1C	109.3	H17A—C17—H17B	109.5
C11—C1—H1C	109.3	C14—C17—H17C	109.5
O1—C1—H1D	109.3	H17A—C17—H17C	109.5
C11—C1—H1D	109.3	H17B—C17—H17C	109.5
H1C—C1—H1D	107.9	C22—C21—C26	117.9 (3)
O2—C2—O1	122.3 (3)	C22—C21—C2	120.1 (3)
O2—C2—C21	124.5 (3)	C26—C21—C2	122.0 (3)
O1—C2—C21	113.2 (3)	C23—C22—C21	121.0 (3)
C16—C11—C12	117.5 (3)	C23—C22—H22	119.5
C16—C11—C1	120.8 (3)	C21—C22—H22	119.5
C12—C11—C1	121.7 (3)	C22—C23—C24	120.8 (3)
C13—C12—C11	121.1 (3)	C22—C23—H23	119.6
C13—C12—H12	119.5	C24—C23—H23	119.6
C11—C12—H12	119.5	N1—C24—C23	121.2 (3)
C14—C13—C12	121.3 (3)	N1—C24—C25	120.1 (3)
C14—C13—H13	119.4	C23—C24—C25	118.6 (3)
C12—C13—H13	119.4	C26—C25—C24	120.7 (3)
C13—C14—C15	117.3 (3)	C26—C25—H25	119.6
C13—C14—C17	120.7 (3)	C24—C25—H25	119.6
C15—C14—C17	122.0 (3)	C25—C26—C21	121.0 (3)
C16—C15—C14	121.3 (3)	C25—C26—H26	119.5
C16—C15—H15	119.4	C21—C26—H26	119.5
C14—C15—H15	119.4		
			
C2—O1—C1—C11	94.1 (4)	O2—C2—C21—C22	−0.9 (4)
C1—O1—C2—O2	−1.9 (5)	O1—C2—C21—C22	179.1 (3)
C1—O1—C2—C21	178.1 (3)	O2—C2—C21—C26	177.6 (3)
O1—C1—C11—C16	95.1 (4)	O1—C2—C21—C26	−2.4 (4)
O1—C1—C11—C12	−85.7 (4)	C26—C21—C22—C23	0.7 (4)
C16—C11—C12—C13	0.8 (5)	C2—C21—C22—C23	179.2 (3)
C1—C11—C12—C13	−178.5 (4)	C21—C22—C23—C24	0.0 (4)
C11—C12—C13—C14	−0.8 (5)	C22—C23—C24—N1	−178.3 (3)
C12—C13—C14—C15	0.7 (5)	C22—C23—C24—C25	−0.7 (4)
C12—C13—C14—C17	−179.2 (4)	N1—C24—C25—C26	178.2 (3)
C13—C14—C15—C16	−0.6 (5)	C23—C24—C25—C26	0.6 (4)
C17—C14—C15—C16	179.3 (4)	C24—C25—C26—C21	0.2 (4)
C14—C15—C16—C11	0.7 (6)	C22—C21—C26—C25	−0.8 (4)
C12—C11—C16—C15	−0.7 (5)	C2—C21—C26—C25	−179.3 (3)
C1—C11—C16—C15	178.5 (4)		

### Hydrogen-bond geometry (Å, °)

**Table:**

*D*—H···*A*	*D*—H	H···*A*	*D*···*A*	*D*—H···*A*
N1—H1A···O2^i^	0.88 (4)	2.12 (5)	2.977 (4)	164 (4)
N1—H1B···N1^ii^	0.96 (6)	2.37 (6)	3.278 (3)	158 (4)

Symmetry codes: (i) *x*−1, *y*, *z*; (ii) −*x*, *y*−1/2, −*z*+2.
